# Analysis of Spatio-Temporal Characteristics of Urban Economic Resilience and Influencing Factors in Guangdong-Hong Kong-Macao Greater Bay Area

**DOI:** 10.3389/fpubh.2022.922096

**Published:** 2022-06-28

**Authors:** Yuanyuan Zhang, Zehui Chen, Bo Tang, Hua Sun

**Affiliations:** ^1^School of Resources and Planning, Guangzhou Xinhua University, Guangzhou, China; ^2^Faculty of Innovation and Design, City University of Macau, Macau, China

**Keywords:** economic resilience, public health emergencies, PSR model, Guangdong-Hong Kong-Macao Greater Bay Area, spatial and temporal evolution

## Abstract

Due to the changes in the domestic and international economic situation in the post-pandemic era, the economic development of the Guangdong-Hong Kong-Macao Greater Bay Area has become unstable in many aspects. The paper adopted the Pressure-State-Response (PSR) model to build a regional economic resilience evaluation system from the perspective of public health emergencies. Then, the spatial and temporal evolution of the economic resilience of the Guangdong-Hong Kong-Macao Greater Bay Area and the influencing factors were explored by using entropy weight method, GIS and gray correlation method. The conclusions show that: (1) Temporally, the economic resilience of the Guangdong-Hong Kong-Macao Greater Bay Area has generally increased from 2010 to 2021, and is divided into three main stages: rapid development, adjustment to fluctuations and stable development. (2) Spatially, the overall pattern of economic resilience in the Guangdong-Hong Kong-Macao Greater Bay Area is high in the middle and south and low in the northwest, and shows a “stochastic—equalized—polarized” pattern of transformation. (3) In terms of influencing factors, economic status and economic response are the main dimensions affecting the resilience level of the economic system in the Guangdong-Hong Kong-Macao region. The level of scientific research and innovation, medical governance, government regulation and the rationalization of the industrial system are the key factors.

## Introduction

The COVID-19 epidemic swept the world in 2020, which caused a number of problems including disruption of international trade, price fluctuations, increased unemployment, and increased pressure on government spending on health care, thereby impacting the economic stability of countries. The intensification of globalization has made the world economy a mutually integrated organic whole, but at the same time, the economic development of cities and regions is inevitably influenced by the external environment. With the increasingly frequent flow of products, resources, and human resources between countries, the threat of global epidemics will be a problem that cities and regions must deal with in their economic development, and economic resilience has also become a strategic issue that countries must pay attention to in their economic development (1). Economic resilience is the ability of an economic system to recover from shocks and disruptions (2), focusing on the ability of the region to resume production, escape from the economic downturn crisis and recover successfully after suffering from external shocks. Located at the frontier of the coastal opening area in China, the Guangdong-Hong Kong-Macao Greater Bay Area is one of the most active and open areas in China for foreign trade, as well as a strategic place for China to enhance the innovation and competitiveness of the national economy. The cultivation of its economic resilience is of great significance for the healthy and stable development of China's regional economy. In addition, because of the high openness of the Guangdong-Hong Kong-Macao Greater Bay Area, it is more vulnerable to the influence of the external environment than other regions, and the study of its economic resilience also provides a valuable reference for other cities and regions. In this paper, urban economic resilience in the Guangdong-Hong Kong-Macao Greater Bay Area is studied from the perspective of public health emergencies, and the evaluation system of urban economic resilience is improved and the related influencing factors are examined.

Numerous studies have shown that population health and infectious diseases are strongly associated with regional economic growth. In earlier years, Bloom et al. (3) found a significant positive effect of population health on macroeconomic growth by studying the micro effects of health on wage levels; the outbreak of international public health emergencies can have a significant impact on the global economy, leading to significant declines in urban industrial output and GDP (4), declines in the real estate sector, market price fluctuations (5) and global economic contraction (6). Especially in the case of outbreaks of infectious diseases such as SARS and the COVID-19 epidemic, “lockdown” and “quarantines” are required for cities. Deb et al. (7) found that these measures would result in a loss of 15% of the monthly average industrial output by looking at daily economic data. At the same time, many scholars have conducted a series of studies on how to deal with and reduce the economic impact of such public health emergencies. In particular, after the outbreak of the COVID-19 epidemic, scholars in many countries have actively explored ways to reduce the impact of the epidemic on urban economies from the perspectives of government public investment decisions (4), policy orientation (8) and medical resource availability (9). Chinese scholars such as Li (10), Liu and Li (11), and Qin and Liu (12) have used resilience theory to analyze the resilience of economic systems in cities and regions from the perspectives of regional economic linkages, industrial structures and knowledge innovation capacity (13). The COVID-19 epidemic has enabled scholars to ponder over economic resilience in a more comprehensive way.

Regional economic resilience refers to a region's economic resilience to resist market competition or environmental shocks and recover its growth path (14), which is mainly composed of four processes: vulnerability, resistance, stability, and resilience (15). Economic resilience can better explain the process of self-regulation and change of economic systems under external conditions. However, due to the inherent complexity and geographical variability of economic systems, there is no unified system of indicators to assess them. At present, the research on regional economic resilience mainly includes two aspects: influencing factors and resilience evaluation. Studies on the influencing factors range from the impact of national environment (16), policies and institutions (17), regional linkages (18), industrial structure and multiculturalism (19) on resilience. Most scholars have evaluated the structure of the regional economic system and its vulnerability and resilience to the crisis, the degree of recovery from the recession, and the redevelopment and renewal capacity of the economic system after the shock (20), with the resilience and recovery capacity being the most emphasized research points (21). Therefore, regional economic resilience measurement methods can be broadly divided into two categories: one is to measure a core variable as a reflection of economic resilience. Zhu (22) analyzed China's economic resilience and dynamism through Purchasing Managers' Index (PMI), and concluded that the key to economic development is to drive domestic demand with investment and consumption, to pull back the economic function with the service industry, to drive exports with the policy of stabilizing foreign trade and foreign capital, and to promote the transformation and upgrading of the manufacturing industry with new dynamic energy. The other is to establish a system of comprehensive indexes for assessment. Hu Xiaohui and other Chinese scholars believe that regional economic resilience should contain three characteristics: complex adaptability, non-equilibrium and dynamic evolution. They also deduced four connotations from the characteristics, including resilience, recovery capacity, reorganization capacity and renewal capacity (23). Cai Jianming (24) sorted out the conceptual differences in ecological resilience, engineering resilience, economic resilience and social resilience. Xu Yuanyuan concluded that uncorrelated diversity and innovation are the core of a region's economic resilience level by building a regression analysis model. Meanwhile, she chose the spatio-temporal bifixed Durbin model and concluded that science and technology, transportation development, and fixed asset investment have strong direct effects on regional economic resilience (25). Shao and Zhu (26) found that regions and cities with high level of development and strong professional production capacity suffered more drastic economic shocks from the epidemic, but these places could display stronger resilience with close regional connectivity and information technology.

In summary, the current analysis of economic resilience is mainly carried out from two aspects, namely, capital market and regional industrial structure, to explain and analyze the laws and influencing factors of economic resilience, but there are still some shortcomings in research perspectives and research methods. Although the current evaluation system of economic resilience theory is richly constructed, most of the studies focus on the impact of natural disasters or financial crises on the region, and the analysis of the close correlation between public health emergencies and the economy is not enough. At the same time, the dynamic pattern and influencing factors of economic resilience can be further analyzed. Therefore, this paper adopts the Pressure-State-Response (PSR) model to build a regional economic resilience evaluation system. Moreover, the entropy power method, GIS and gray correlation method are combined to explore the spatial and temporal evolution and influencing factors of the economic resilience of Guangdong-Hong Kong-Macao Greater Bay Area, so as to provide suggestions for the economic transformation and upgrading of the Guangdong-Hong Kong-Macao Greater Bay Area under the post-pandemic era.

## Research Methodology and Data Sources

### Theoretical Framework

The PSR model was originally proposed by Canadian statisticians David and Tony to address ecological and environmental issues (27). It reveals a direct chain of interactions between the influencing factors, the system, and human activities, and emphasizes the intrinsic mechanism of frequent material and energy cycles between the system and human activities (28). Economic resilience is the ability of a system to adapt, resist, recover and develop in response to external disturbances. In the system, it first experiences risky shocks, then keeps itself stable after the shocks, and finally transforms the crisis into an opportunity to achieve the innovative development. Therefore, strong economic resilience can be seen as a spiral upward process in which economic systems continuously adapt to risks and recover and strengthen themselves. The resilience of the regional economic system from the perspective of public health also has such process property, which means that the region will experience the dynamic process of “stabilization, shock and re-stabilization” after the outbreak and disturbance of public health emergencies and under the stimulus input.

At present, most of the processes of economic resilience evaluation still decompose the regional system into parallel vertical subsystems such as industrial structure, technology level and economic scale, but the positive and negative feedback processes between the front-end and the regional subject are still less emphasized. If the PSR model is used, it is possible to describe more accurately the process properties of the economic resilience of the system by using the logical thinking of mutual regulation of the region from the subject system and the stress risk input to the system and the three-way feedback of the system's own state changes.

**Figure 1:**
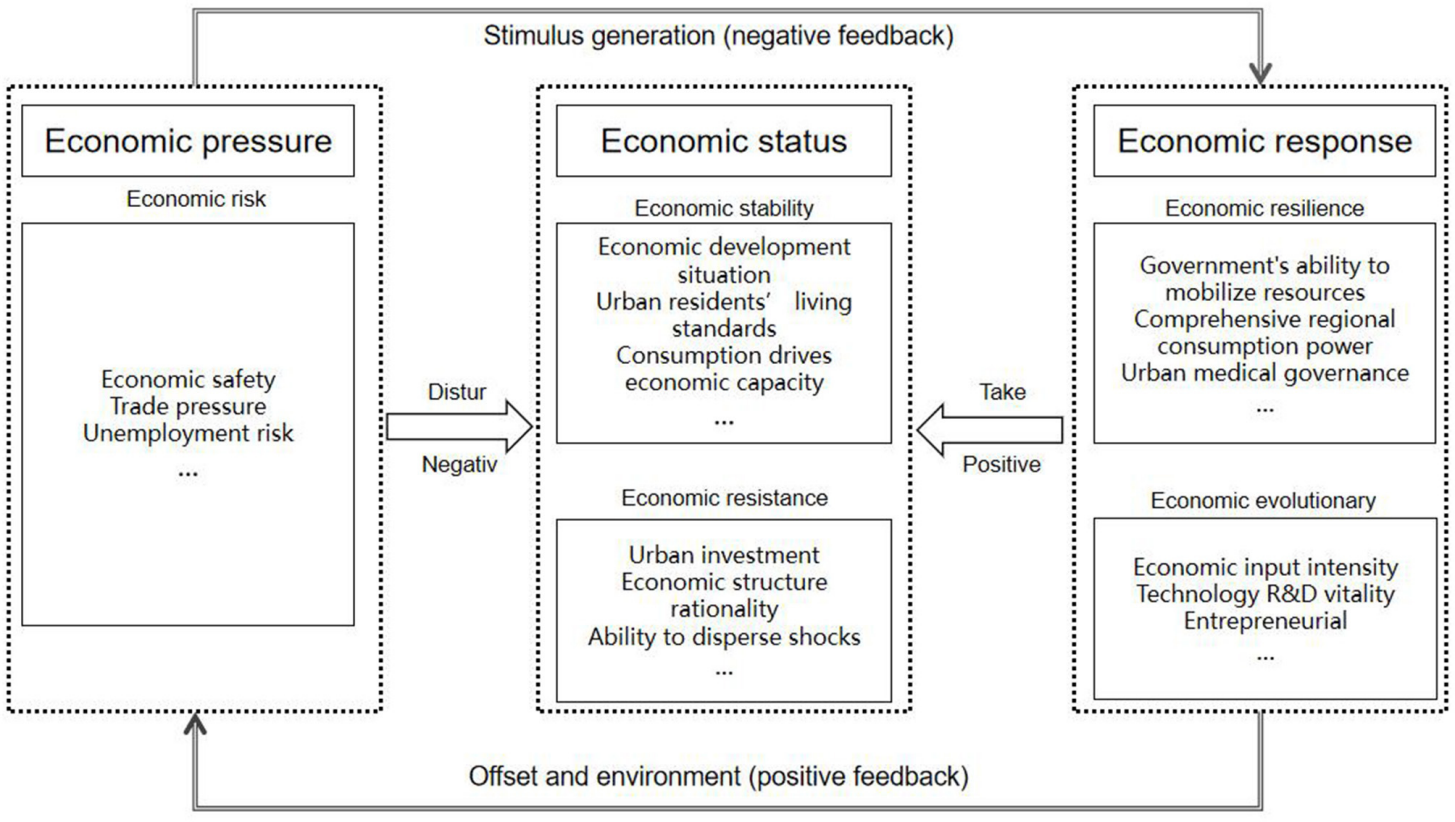
Theoretical framework.

Therefore, based on the above understanding, the economic system process can be divided into three stages: riskiness before the shock, stability and resistance during the shock, and resilience and evolutionary power after the shock, which correspond to the three process elements of “pressure-state-response” in the PSR model, respectively. In this study, three dimensions of the resilience process are proposed for the regional economic system to cope with the disturbance, namely, economic pressure, economic status, and economic response as shown in Figure 1.

Economic pressure refers to the pressure on the system during the outbreak of public health emergencies and its aftermath, and is characterized by its own potential riskiness. It can be interpreted as the low exposure of the system to the risk of public health emergencies and its negative effects, reflecting the external disturbances and endogenous perturbation dynamics of the regional economic system. Economic vulnerability can be used as a measure of the relative loss value of the regional economy, usually manifested as employment decline, trade pressure, and financial risk, that is, risk vulnerability (29).

Economic status refers to the stability of the system's own structure and its resistance to disturbances, while it is constantly changing in the process of negative effect of “pressure” and positive feedback of “response.” Economic status consists of economic stability and economic resistance. Economic stability can spontaneously maintain the ability of its own structural system to function as an internal stabilizer; economic resistance refers to the ability of the regional economic system to withstand shocks. It usually operates in the form of consumption level, economic base, industrial structure and capital input.

Economic response refers to the ability of the subjects of the urban economic system to respond and take actions in response to disturbances, reflecting the ability of the subjects of the regional economic system to deal with risks, learn from shocks and disturbances and optimize their own organizational structure. Economic recovery capacity means that the speed and extent of recovery from shocks can be achieved through rapid and diversified response measures. Economic evolutionary power is the ability to renew and reorganize internal structures and functions, generating new development models and paths for the purpose of strengthening the development of economic agents.

### Assessment System

For the connotation of economic resilience, Martin proposed four dimensions of regional economic vulnerability, resistance, stability, and resilience to break down the operation process of economic resilience. Zeng (30) also constructed a comprehensive index system of regional economic resilience from three different dimensions of evolutionary power, resistance and resilience. Zhang (31) analyzed the four aspects of regional economic stability, regional economic diversity, regional economic innovation capacity, and regional economic vitality. Based on this, an index system of economic resilience of the Guangdong-Hong Kong-Macao Greater Bay Area is constructed from three dimensions of economic pressure, economic status and economic response, as shown in Table 1.

**Table 1:** Table of regional economic resilience indexes.

**System level**	**Criterion level**	**Index level**	**Index definitions**	**Nature**	**Index calculation**	**Weight**
Economic pressure	Economic risk	X_1_ foreign trade dependence (%)	Urban trade pressure	–	Total import and export trade/GDP	0.0539
		X_2_ urban registered unemployment rate (%)	Urban unemployment risk	–	Number of unemployed persons/(number of employed persons + number of unemployed persons)	0.0310
		X_3_ loan-to-deposit ratio (%)	Economic security	^*^	Ending balance of various loans/Ending balance of various deposits	0.0792
Economic status	Economic stability	X_4_ ratio of total retail sales of consumer goods to GDP (%)	The ability of consumption to drive economic growth	+	Total retail sales of social consumer goods/GDP	0.0468
		X_5_ urban per capita disposable income(yuan)	Living standard of urban residents	+	Total urban income/resident population	0.0418
		X_6_ local GDP (in 10,000 yuan)	Economic development status	+	Yearbook statistics	0.0550
	Economic resistance	X_7_ ratio of tertiary industry output to GDP (%)	Reasonable degree of economic structure	+	Tertiary industry output/GDP	0.0700
		X_8_ ratio of fixed asset investment to GDP (%)	The level of urban investment	+	Fixed asset investment/GDP	0.0628
		X_9_ industrial value added (in 100,000,000 yuan)	Industrial situation	+	Yearbook statistics	0.0620
		X_10_ diversification of industrial structure	Ability of the city to disperse economic shocks	+	Diversification index (DIV)	0.0702
Economic response	Economic recovery capacity	X_11_ general public budget revenue (in 10,000 yuan)	Government's ability to mobilize resources	+	Yearbook statistics	0.0494
		X_12_ ratio of health expenditure to fiscal expenditure	Scale of government funding for epidemic prevention	+	Health spending/financial spending	0.0455
		X_13_ number of hospitals	Urban medical governance capacity	+		0.0496
		X_14_ number of public health personnel per 10,000 people		+	(Number of health workers/resident population) ^*^10000	0.0384
		X_15_ number of hospital beds per 10,000 people		+	(Number of beds/resident population) ^*^10000	0.0469
	Economic evolutionary power	X_16_ number of patent applications per 10,000 people (in piece)	Technology R&D vitality	+	Patent Statistics	0.0645
		X_17_ ratio of R&D expenditure to total GDP (%)	Economic investment intensity	+	R&D expenditure/GDP	0.0464
		X_18_ number of patents granted per 10,000 employed population (in piece)	Entrepreneurial innovation spirit	+	Number of patents granted / Total number of employed persons	0.0866

+ is a positive index, − is a negative index, and ^*^ is a moderate index.

### Entropy Method

In order to eliminate the influence caused by data units and subjective determination of index weights, this paper adopts the extreme value entropy method to dimensionlessly process and assign weights to the selected indexes. The greater the index dispersion, the greater the entropy value, and the greater the effective information reflected. It means that the greater the influence on the evaluation, the more significant the weight is. On the contrary, the smaller the index dispersion, the smaller the influence and the smaller the weight (32, 33). The specific formula is shown as follows:

(1) Building the original matrix

Build the matrix of *n* (year/city) ^*^ m (index). Let the value of the jth index for the ith year (city) be *X*_*ij*_.

(2) Standardizing the data

Assuming that the evaluation index *X*_*j*_ is a positive index or negative index, the following can be derived:


(1) $$\begin{array}{l} Positive\;index:\;X_{ij}{}^{*}=\frac{Xij-\min\left\{X_{j}\right\}}{\max\left\{X_{j}\right\}-\min\left\{X_{j}\right\}}\\ Negative\;index:\;X_{ij}{}^{*}=\frac{\max\left\{X_{j}\right\}-Xij}{\max\left\{X_{j}\right\}-\min\left\{X_{j}\right\}} \end{array}$$

If the evaluation index *X*_*j*_ is a suitability index, the following can be derived:


(2) $$\begin{array}{l} X_{ij}{}^{*}=2\frac{\max\left\{X_{j}\right\}-Xij}{\max\left\{X_{j}\right\}-\min\left\{X_{j}\right\}}(\bar{X_{j}}-Xij<\max\left\{X_{j}\right\})\\ X_{ij}{}^{*}=0(Xij\geq \max\left\{X_{j}\right\}Xij\leq \min\left\{X_{j}\right\})\\ X_{ij}{}^{*}=2\frac{Xij-\min\left\{X_{j}\right\}}{\max\left\{X_{j}\right\}-\min\left\{X_{j}\right\}}(\min\left\{X_{j}\right\}<Xij<\bar{X_{j}}) \end{array}$$

(3) Calculate the ratio _*P*_*i*_*j*_ of the ith city/year to the jth index


(3) $$\begin{array}{r} P_{ij}=\frac{{X_{ij}}^{*}}{\sum_{i=1}^{n}{X_{ij}}^{*}} \end{array}$$

(4) Calculate the entropy value *e*_*j*_ of the jth index


(4) $$\begin{array}{r} e_{j}=-\frac{1}{\ln\;n}\sum_{i=1}^{n\sum_{ij}e_{j}}P_{ij}\ln \end{array}$$

(5) Calculate the co-efficient of variation *d*_*j*_ of the jth index


(5) $$\begin{array}{r} d_{j}=1-e_{j} \end{array}$$

(6) Calculate the weight *w*_*j*_ of the jth index


(6) $$\begin{array}{c} w_{j}=\frac{d_{i}}{\sum_{j=1}^{m}\;d_{i}}(j=1,2,...,m) \end{array}$$

### Gray Correlation Analysis

Based on the data samples, the gray correlation analysis uses the geometric similarity between the parent sequence and the signature sequence to identify the closeness of the association, and obtains the gray correlation between the parent sequence and each signature sequence. The greater the correlation, the closer the relationship between the signature sequence and the parent sequence, and vice versa. The formula is as follows:


(7) $$\begin{array}{r} \zeta i\left(j\right)=\frac{\overset{\min}{i}\overset{\min}{j}|Q_{i}-{x^{{}^{\prime }}}_{i}\left(j\right)|+\rho^{*}\overset{\max}{i}\overset{\max}{j}|Q_{i}-{x^{{}^{\prime }}}_{i}\left(j\right)|}{\left|Q_{i}-{x^{{}^{\prime }}}_{i}\left(j\right)|+\rho^{*}\overset{\max}{i}\overset{\max}{j}|Q_{i}-{x^{{}^{\prime }}}_{i}\left(j\right)\right|},\left(i=1,2,...,n\right) \end{array}$$

where, ζ_i_(j) denotes the gray correlation co-efficient, Q_i_ denotes the parent sequence, ${\text{x}}_{\text{i}}^{\prime }$(j) denotes the signature sequence, ρ denotes the identification co-efficient, with ρ being 0.5. The correlation co-efficient is the correlation value between the signature sequence and the parent sequence at each moment. Due to the multi-valued nature of the correlation co-efficient, it is difficult to make an overall comparison, so the average value of the correlation co-efficients at all moments is used as the value of the correlation between the comparison sequence and the reference sequence. The calculation formula is as follows:


(8) $$\begin{array}{r} r_{j}={\frac{1}{n}}^{*}W_{j}^{*}\sum_{i=1}^{n}\zeta_{i}\left(j\right),i=1,2,...,n;j=1,2,...,m \end{array}$$

where, *n* denotes the number, W_j_ denotes the weight. The greater the value of the correlation co-efficient *r*_*j*_, the higher the impact of the index on the comprehensive resilience level.

### Data Source

**Figure 2:**
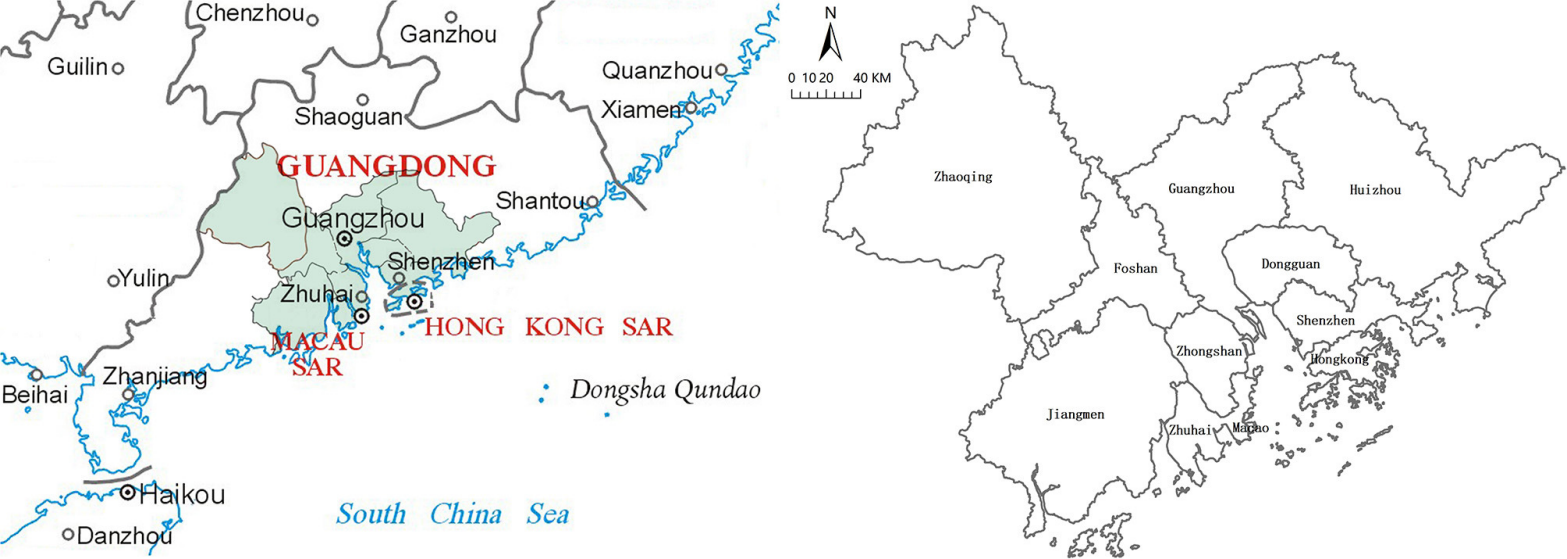
Overview of the study area. Source: A standard map of the standard map service system of the Ministry of Natural Resources [Review No.: No. GS(2019)4342].

Located in the south of China, the Guangdong-Hong Kong-Macao Greater Bay Area now covers Guangzhou, Shenzhen, Foshan, Dongguan, Zhuhai, Huizhou, Zhongshan, Jiangmen, Zhaoqing, as well as Hong Kong and Macau as shown in Figure 2. The Greater Bay Area is densely populated with an export-oriented economy. Moreover, it has developed comparative and comprehensive advantages in technology, finance, foreign trade, transportation and tourism, and is one of the two major economic engines of China.

The time span of this study is 2010–2019, and the data sources include the fourth national economic census, statistical yearbooks of each region, statistical yearbooks of the Pearl River Delta city cluster, statistical yearbooks of cities in China and national economic statistical bulletins. Since the statistical standards vary from city to city, the number of registered unemployed in Hong Kong and Macau in this study is the unemployed population, the total retail sales of social consumer goods in Hong Kong is the total value of retail sales, and the total retail sales of social consumer goods in Macau is the total sales of retail trade. The total volume of imports and exports in Hong Kong and Macao is the total volume of trade; at the same time, all of them are converted into RMB at the current exchange rate of the year.

## Temporal and Spatial Evolution Characteristics of the Guangdong-Hong Kong-Macao Greater Bay Area

### Temporal Evolution: Increasing Overall Volatility, With Distinct Characteristics in Phases

**Figure 3:**
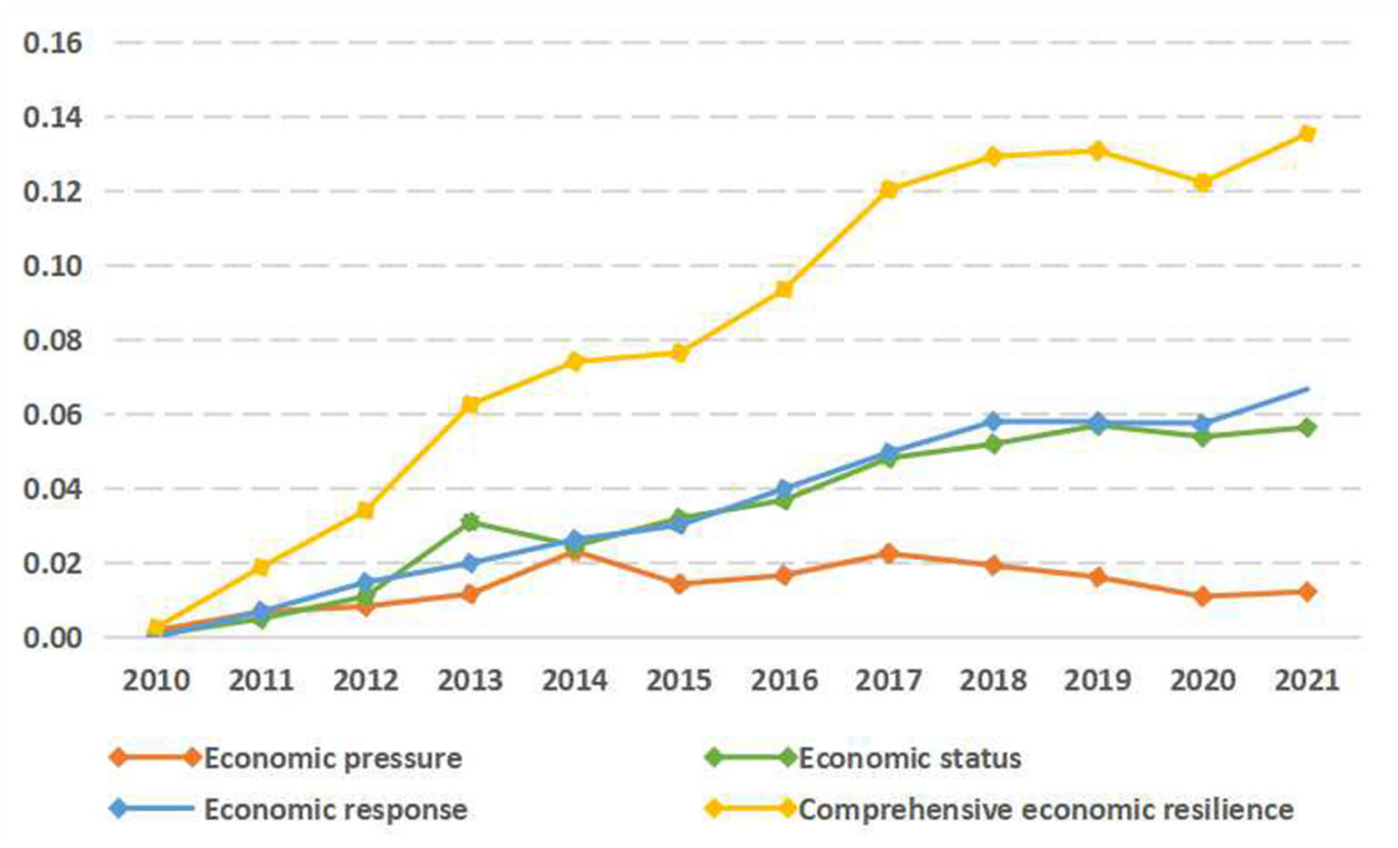
Temporal evolution of the overall economic resilience of the Guangdong-Hong Kong-Macao Greater Bay Area.

The economic data of the Greater Bay Area from 2010 to 2021 are used to construct a matrix of year^*^ index, and the results are shown in Figure 3. The economic resilience level of the Greater Bay Area has generally improved, except for a small decline in the regional economic resilience in 2020. It is divided into three main phases by the development trajectory:

First, the period from 2010 to 2013 represents the period of rapid development of the Greater Bay Area. During this period, the Greater Bay Area advocated a model of rapid economic construction, thus maintaining a high rate of economic resilience development, with economic pressure, economic status and economic response all making improvements to varying degrees. After the 2009 H1N1 and 2008 financial crisis, economic pressure, economic status and economic response maintained a dynamic equilibrium relationship that promotes each other. At this time, the economic pressure risk is high, the economic status foundation is weak, and the economic response capability is insufficient. If one of them fails, the whole system will be affected. Therefore, they are developing more in a relatively “conservative” way. In terms of a single index, fixed asset investment and the deposit-to-loan ratio have a short-lived mini-peak, which caters to the massive tides of economic development in the early stages and a huge amount of capital is needed to promote the economic vitality of the Greater Bay Area.

Second, the period from 2013 to 2017 represents the period of fluctuations and adjustment of economic resilience. It is the transition period of industrialization in the Greater Bay Area, where economic pressure and economic status underwent rapid changes and adjustments, and then the three converged in 2014, and then became differentiated. Economic pressure, economic status, and economic response became distant from each other, and the economic system of the Greater Bay Area regulated itself mainly in the form of status-response. From a single index, the value added of tertiary industry witnessed explosive growth, the economic structure of the Greater Bay Area was upgraded, entrepreneurship and scientific research vitality emerged in the budding stage of development, and the industrial structure tended to be diversified and intelligent.

Third, the period from 2017 to 2021 represents the period of stable development of economic resilience. At this point, the industrial transformation of the Greater Bay Area matured, from the original high-speed development mode to high-quality development mode, and the industrial structure was gradually adjusted from labor-intensive to knowledge-intensive industries. Economic pressure gradually decreased, while economic status and economic response slowly increased, and the distance between the three gradually expanded, with the economic system in a high-exposure development model. The fall in economic pressure means that the economic system is more likely to face high-risk shocks. However, the economic system is less likely to collapse even after a public health emergency by continuously strengthening the substrate of economic status, increasing the threshold of economic resilience against the maximum risk intensity, and establishing an economic response at the same time. Furthermore, the rapid response and regulation allows the economic system to return to its original stage of development, as exemplified by the COVID-19 epidemic in 2020. The economic resilience of the Greater Bay Area declined briefly when the crisis occurred, but soon rebounded and continued to grow. In terms of a single index, the rapid rise in entrepreneurial innovation and R&D vitality signifies the beginning of a massive renewal of the industrial structure based on technological innovation. The spike in the loan-to-deposit ratio could easily cause a payment crisis in banks, which would spread further to lead to a financial crisis.

**Figure 4:**
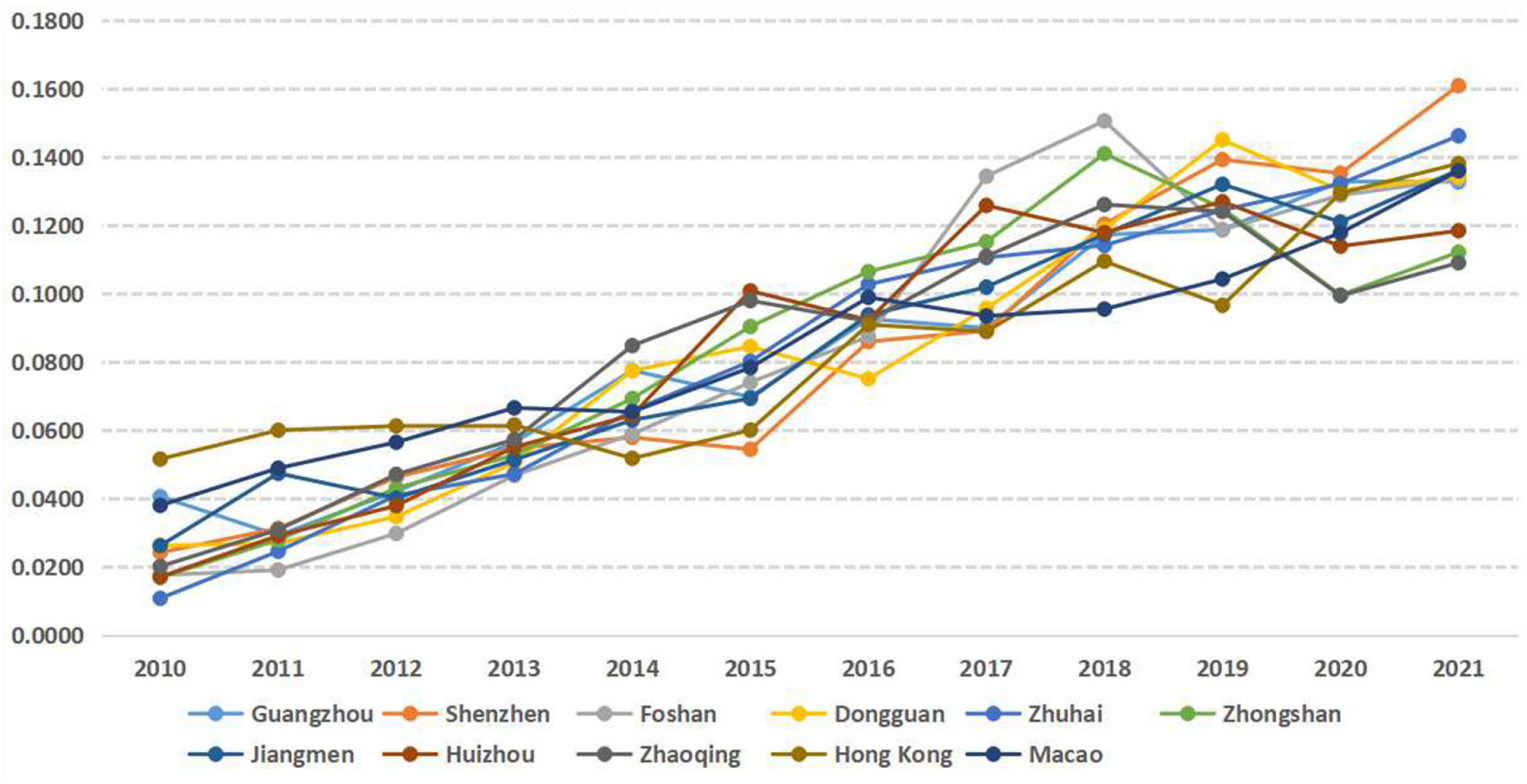
Sequence diagram of economic resilience of cities in the Guangdong-Hong Kong-Macao Greater Bay Area.

**Figure 5:**
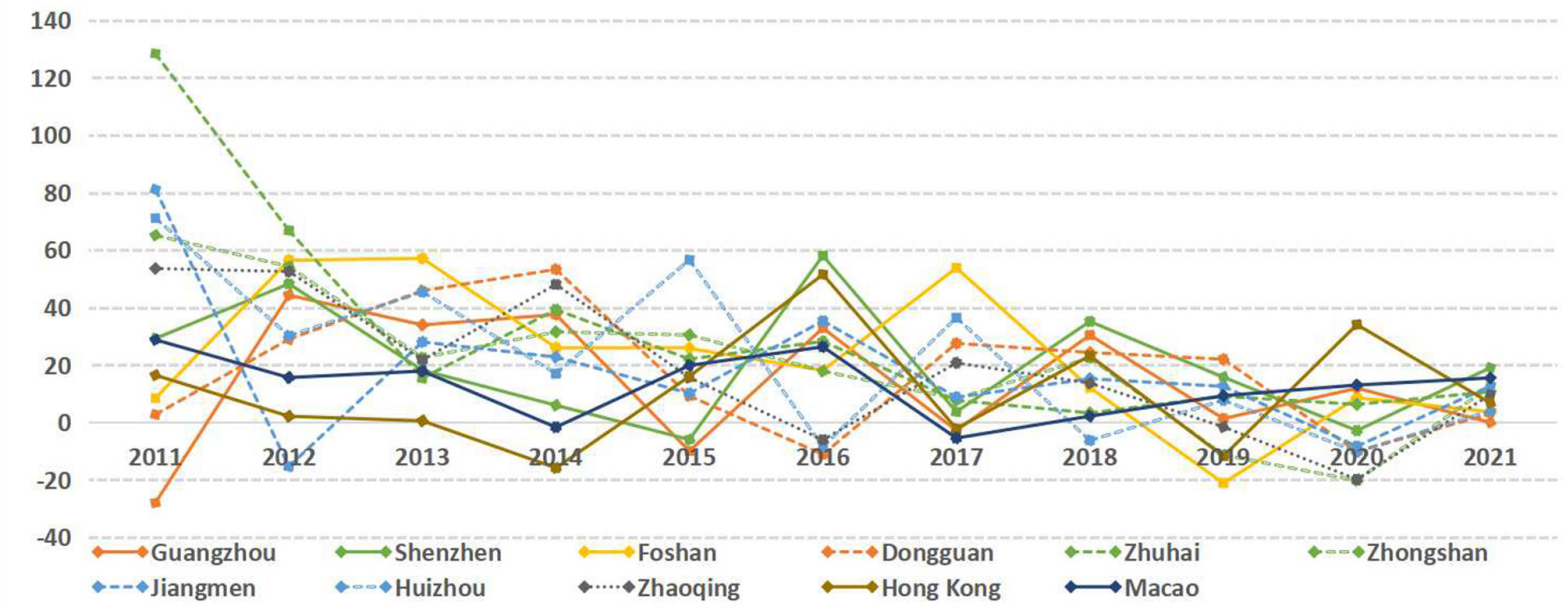
Rate of change in economic resilience of cities in the Guangdong-Hong Kong-Macao Greater Bay Area.

A matrix of year^*^ index is constructed from the economic data of 11 cities in the Greater Bay Area of Guangdong-Hong Kong-Macao, and the results are shown in Figure 4. The economic resilience of each city in the Greater Bay Area has been improved in general. The average value of the growth rate of the 11 cities is used as the threshold value, and they are classified into two categories: rapid development and slow development:

First, the cities with rapid development include Zhuhai, Foshan, Huizhou, Zhongshan, and Shenzhen. Among them, Zhuhai has the fastest growth in economic resilience in the past 12 years, with an average growth rate of 30.61% as shown in Figure 5. Most of the cities in this category are dominated by high-tech manufacturing and other emerging industries, such as Zhuhai (biomedical), Shenzhen (Internet), Foshan (advanced manufacturing), Huizhou, and Zhongshan (electronics);

Second, the cities with slow development include Zhaoqing, Jiangmen, Dongguan, Guangzhou, Macau, and Hong Kong. Among them, Hong Kong is the slowest in building economic resilience, with a rate of 10.95% as shown in Figure 5. Most of the cities in this category are highly involved in global economic networks, such as Hong Kong (transit trade and finance), Macau (gaming and finance), Dongguan and Guangzhou (export trade). Although cities with high involvement in the global economy facilitate industrial division of labor and economic development to some extent, the flow of goods and population is restricted and the economic system is easily affected when faced with global public health emergencies such as H1N1, H7N9 and the COVID-19 epidemic (34). The other part is due to their weak economic base, such as Jiangmen and Zhaoqing (raw material processing).

### Spatial Distribution: A Pattern of High in the Central and Southern Parts and Low in the Periphery, Evolving in a “Stochastic—Equalized—Polarized” Pattern

**Figure 6:**
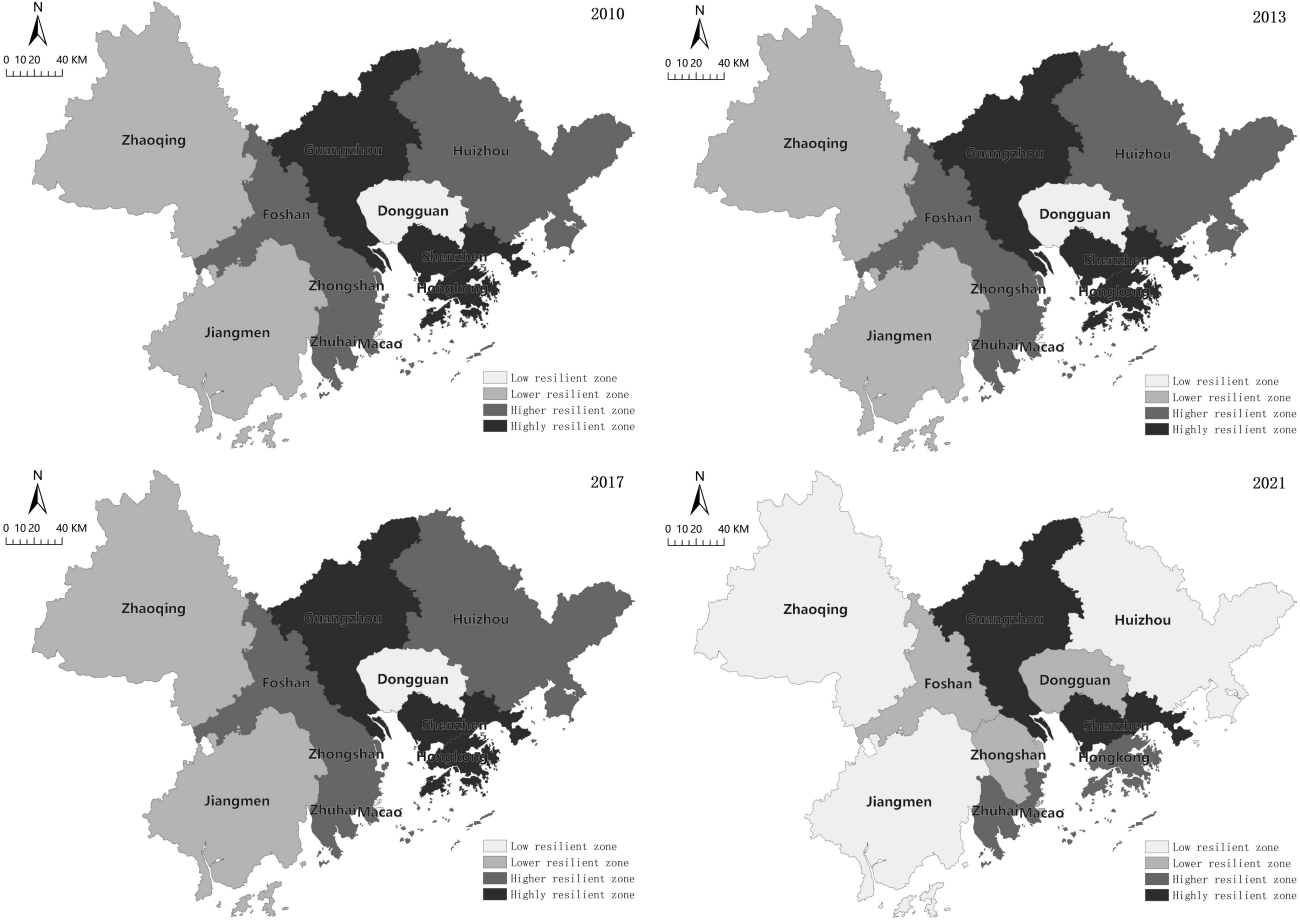
Spatial distribution of economic resilience in the Guangdong-Hong Kong-Macao Greater Bay Area.

A matrix of city^*^ index is constructed by counting the panel data of each city in the Guangdong-Hong Kong-Macao Greater Bay Area from 2010 to 2021. Taking the average value of economic resilience of the cities in the Greater Bay Area from 2010 to 2021 as the criterion, four representative nodes were selected in 2010, 2013, 2017 and 2021. With the help of natural breaks classification method in GIS (35), the regional economic resilience is divided into four types: low resilience zone, relatively low resilience zone, relatively high resilience zone and highly resilient zone. As shown in Figure 6, the spatial layout characteristics have a large variation in different periods, and its characteristics mainly present a pattern of high in the central and southern parts and low in the periphery, and its distribution shifts along the stochastic—equalized—polarized pattern.

First, the spatial pattern of the high resilience zone is stable. The high resilience zone is mainly concentrated in the first-tier urban areas, roughly in the central and southeastern parts of the Greater Bay Area. Initially, Guangzhou, Shenzhen and Hong Kong were the two poles of resilience, and then Guangzhou and Shenzhen became the two poles. Guangzhou benefits from the advantages of gross domestic product, industrial value added, fiscal revenue and hospitals. Guangzhou, as the capital city of Guangdong Province, can carry out division of labor and cooperation within the provincial cities, promote technology transfer and industrial upgrading, form industrial linkage effects, and optimize industrial structure (36), which can disperse most of the economic shocks and strengthen its economic status resilience. Shenzhen mainly benefits from its gross domestic product, industrial value added and technological R&D emphasis, and changes from a labor-intensive industrial model dependent on Hong Kong to a knowledge-intensive industrial model with independent innovation. As a result, a full set of high-tech industrial chain of “R&D-production” is formed in combination with industrial bases to realize spillover benefits of knowledge economy (37). Its economic resilience is characterized by the flexible economic evolutionary power to consolidate its economic status, so as to quickly update to cope with the next round of economic crisis.

Second, the space with relatively high resilience shifts. Initially distributed in Jiangmen and Zhongshan in the west wing of the Greater Bay Area, it was subsequently shifted to Hong Kong and Zhuhai in the south of the Greater Bay Area. Benefiting from the advantages of fixed asset investment and entrepreneurship, Zhuhai has generated a wave of investment oriented to scientific and technological innovation, and developed toward the scientific and technological innovation center in the west wing of the Greater Bay Area, thus enhancing the resilience and evolutionary power of economic development. As a global financial center, Hong Kong has a large-scale economy, extensive capital markets, and a well-developed global network, so it has the advantages of gross domestic product, tertiary industry, and per capita disposable income. However, Hong Kong is not backed by local manufacturing industry and is overly dependent on global financial and trade networks, so Hong Kong will be subject to global logistics blockade, trade disruption or order cancellation in case of an economic crisis (38).

Third, the space with relatively low resilience continues to expand. Initially, it was distributed in Dongguan, Foshan, and Zhuhai, which are between high and relatively high resilience values, and then expanded to the peripheral cities of the Greater Bay Area, and finally returned to Dongguan, Foshan, Zhongshan, and Macau. Like Hong Kong, Macau follows a free port economic policy, and as the only gaming financial city in China, Macau is home to a large amount of non-investment capital, resulting in a high per capita income and a developed tertiary industry, but it is at the bottom of the Greater Bay Area in health care and scientific and technological R&D. In addition, Macau is exposed to a high risk due to the excessive lending (93.20% of the loan-to-deposit ratio in 2020). Once the economic crisis strikes, Macau will be subject to a series of economic chain reactions and the credit crisis will be further aggravated. Although Foshan and Zhongshan do not have as many economic advantages as Guangzhou, Shenzhen, and Zhuhai, they both have their distinctive strengths. Foshan has achieved a good balance in terms of the loan to deposit ratio, and the development model of specialized towns in Foshan has been the unique feature of its economy. Nowadays, Foshan has been trying to diversify the risk by transitioning the original specialization model to a non-related diversification model through banking, scientific and technological innovation and financial means (39). In recent years, Zhongshan has introduced a series of policies to encourage scientific and technological innovation, so it has made progress in entrepreneurial innovation, and in the future, it will implement industrial upgrading and adjustment through economic evolutionary power, just like Shenzhen, so as to improve the level of resilience. Dongguan, like Foshan, started with manufacturing industries, but its spatial proximity to Guangzhou and Shenzhen has allowed it to receive economic spillovers from both cities, resulting in a healthy loan to deposit ratio and vibrant entrepreneurship. However, unlike Foshan, Dongguan is mainly oriented to foreign markets and will be exposed to the same trade risks as Hong Kong.

Fourth, the space with low resilience is basically in the periphery, such as Jiangmen, Zhaoqing, and Huizhou. Except for 2017, when Huizhou rose to a relatively low resilience, it has been in low resilience for the rest years, with poor performance of various economic indexes. Huizhou suffers from a lack of capital and a large population outflow from Huizhou, resulting in a serious aging population, sluggish economic development, and slow recovery in times of economic crisis. Jiangmen's industrial structure is still being shifted from agriculture to industry, and its economic resilience is low due to its low per capita income, unstable economic base, and delayed industrial restructuring. Zhaoqing suffers from low fiscal revenues and inadequate health personnel, and its industrial base is weak, making it extremely difficult to respond to public health emergencies if they occur.

### Influencing Factors: Economic Status and Economic Response as the Key Factors Influencing the Economic Resilience of the Greater Bay Area

The economic resilience value of the Greater Bay Area calculated is used as the parent sequence of the gray correlation analysis, and the other 18 indexes are used as the signature sequence to analyze the fitting effect of the economic resilience with each index, so as to determine its influencing factors. According to Table 2, the average correlation between the economic pressure resilience index and the regional economic resilience level is the lowest, while the economic response resilience index is the highest of the three, followed by the economic status resilience index. It shows that the Greater Bay Area is not subject to much fluctuation of pressure between 2010 and 2021. The impact of pressure resilience on the level of economic resilience of the Guangdong-Hong Kong-Macao Greater Bay Area is relatively low. Moreover, the resilience level of the economic system of the Greater Bay Area depends largely on the resilience level of the economic status and the resilience level of the economic response of the region.

**Table 2:** Gray correlation of criterion levels of economic resilience in the Guangdong-Hong Kong-Macao Greater Bay Area.

**Criterion level**	**Correlation value**	**Correlation degree**
Economic pressure	0.5966	Medium
Economic status	0.6436	High
Economic response	0.6913	High

#### Low Impact of Economic Pressure Resilience, Not the Goal of Economic Resilience Development in the Greater Bay Area

As shown in Table 3, the average correlation of the resilience of economic pressure is 0.5966, which is a medium correlation level. Among them, the correlation co-efficient of the loan-to-deposit ratio is the largest and the correlation co-efficient of foreign trade dependence is the smallest, so the loan-to-deposit ratio is the main factor influencing the economic pressure. From the experience of the development of the greater bay areas worldwide, the development of the capital market has an irreplaceable and important role in the construction of the greater bay area. The Guangdong-Hong Kong-Macao Greater Bay Area has an overall high degree of external economic dependence and a high degree of economic export orientation. However, with the advancement of the integrated development of Guangdong-Hong Kong-Macao Greater Bay Area, it has made great achievements in the integration of the commodity market, the factor market and the service market, which has brought into full play the internal linkage and reciprocal development and reduced the degree of external dependence of the economic development to a certain extent. The increase of the loan-to-deposit ratio has brought some risk pressure to the financial market of the Guangdong-Hong Kong-Macao Greater Bay Area. With the outbreak of the COVID-19 epidemic in 2020, the overall loan ratio of the Greater Bay Area already reached 78.69%, which exceeds the normal credit scale of 75% and is prone to financial risks. Therefore, in the complex and changing international environment, the Greater Bay Area needs to develop an innovative path and pattern of financial development and opening, facilitate the interconnection of financial markets and financial infrastructure, enhance the level of financial service innovation, and prevent cross-border financial risks, so as to effectively enhance the efficiency and resilience of economic development.

**Table 3:** Gray correlation of regional economic pressure resilience in the Guangdong-Hong Kong-Macao Greater Bay Area.

**Primary index**	**Secondary index**	**Average correlation**	**Tertiary index**	**Average correlation**
Economic	Economic	0.5966	Foreign trade dependence (%)	0.5573
pressure	risk		Urban registered unemployment rate (%)	0.6003
			Loan-to-deposit ratio(%)	0.6323

#### Economic Status Resilience With the second Largest Correlation, With the Greatest Impact of Economic Stability

As shown in Table 4, among the status resilience indexes, economic stability has the highest correlation value, which indicates that the change of status resilience is heavily influenced by economic stability. Stable development is the foundation for improving economic resilience, while livelihood issues, the degree of economic development and the optimization of industrial structure are fundamentals for stabilizing development and improving economic resistance. In terms of single indices, the regional consumption level, regional economic development and regional industrial structure all have a significant positive impact on the economic status resilience of the Greater Bay Area. Among them, Guangdong-Hong Kong-Macao Greater Bay Area has a high per capita disposable income in urban areas (an average of RMB 99,612.84) and a high ratio of total retail sales of consumer goods, and the general public has a high consumption potential and capacity. The scale effect of consumption is gradually highlighted as Guangzhou is pushing forward the development as an international consumption center city and Shenzhen as the international consumption hub of the Greater Bay Area. In the context of the double cycle, the Greater Bay Area is stabilizing consumption by stabilizing the subjects to help stabilize economic growth. At the same time, the Greater Bay Area is also actively promoting economic transformation and industrial structure optimization. The Greater Bay Area is undergoing industrial transformation and upgrading at the current stage. For most of the manufacturing-oriented cities in the Greater Bay Area, industry is the underlying foundation of the economy, while the tertiary industry is the core force of the future dynamic transformation, so it is necessary to vigorously develop advanced manufacturing and high-tech industries. These are of great significance to the economic stability and economic resilience of the Greater Bay Area.

**Table 4:** Gray correlation of regional economic status resilience in the Guangdong-Hong Kong-Macao Greater Bay Area.

**Primary index**	**Secondary index**	**Average correlation**	**Tertiary index**	**Average correlation**
Economic status	Economic stability	0.6668	Ratio of total retail sales of consumer goods to GDP (%)	0.6014
			Urban per capita disposable income (yuan)	0.6673
			Local GDP (in 10,000 yuan)	0.7315
	Economic resistance	0.6262	Ratio of tertiary industry output to GDP (%)	0.6075
			Ratio of fixed asset investment to GDP (%)	0.5968
			Industrial value added((in 100,000,000 yuan)	0.6872
			Industrial structure diversification	0.6135

#### Economic Response Resilience With the Highest Correlation, With Economic Evolutionary Power as the Key Factor

As shown in Table 5, among the response resilience indexes, the correlation value of the economic evolutionary power is higher overall, indicating that the Greater Bay Area's economic response resilience is mainly influenced by the economic evolutionary power. In terms of single indexes, the correlation co-efficients of scientific research vitality, entrepreneurial innovation, and government resource mobilization capacity are higher, indicating that the government's ability to manage public health emergencies and technological means in the epidemic also play a crucial role in the socio-economic operation. Scientific and technological innovation is an important regional feature of the Greater Bay Area, and it is essential to jointly build an international scientific and technological innovation center and promote industrial synergy and scientific and technological innovation. In the context of high-quality development, the Greater Bay Area should give full play to the leading role of both entrepreneurship and scientist spirit (40), and encourage more talented people to be involved in building the scientific and technological development pattern of the Greater Bay Area. If economic, social, ecological, demographic, and resource resilience constitute the hard power of urban resilience, then institutional, managerial, and organizational resilience are the soft power of urban resilience (41). As the visible hand, the government plays an indispensable role in resource allocation, institutional innovation, and management capacity. In addition, the government is equipped with strong resource integration and grassroots mobilization capabilities in the allocation of public budget, public infrastructure construction, industrial policy, and social welfare, which can effectively enhance economic resilience.

**Table 5:** Gray correlation of regional economic response resilience in the Guangdong-Hong Kong-Macao Greater Bay Area.

**Primary index**	**Secondary index**	**Average correlation**	**Tertiary index**	**Average correlation**
Economic response	Economic recovery capacity	0.6577	General public budget revenue (in 10,000 yuan)	0.7318
			Share of health expenditure in fiscal expenditure	0.6760
			Number of hospitals	0.6213
			Number of public health personnel per 10,000 persons	0.6454
			Number of hospital beds per 10,000 persons	0.6139
	Economic evolutionary power	0.7474	Number of patent applications per 10,000 persons (piece)	0.8392
			Ratio of R&D expenditure to GDP (%)	0.6696
			Number of patents granted per 10,000 employed persons (piece)	0.7335

## Conclusion and Discussion

### Temporally

The economic resilience of the Guangdong-Hong Kong-Macao Greater Bay Area continues to grow, with most cities experiencing an increase in their resilience levels. From 2010 to 2021, the economic resilience of the Greater Bay Area has generally improved, mainly divided into three main stages: rapid development, adjustment in fluctuations and stable development. The regional economic structure has been evolving steadily, showing a healthy development of a pressure-state-response pattern, in which the economic resilience indexes regulate, circulate and influence each other. Among them, the pressure resilience increases in the fluctuations, and the external disturbance of the system decreases. The status resilience increases rapidly, and the robustness level of the system continues to grow. The response resilience increases in fluctuations, and the response capacity is constantly enhanced. In the development of resilience of cities within the region, except for Hong Kong and Macau, where the level of resilience is decreasing, there is a steady increase in the resilience of most of the other cities, reflecting the strong resilience of the Greater Bay Area as a whole. However, under the disturbance of changes in the global economic environment and public health emergencies, it is necessary to pay close attention to the dynamics of the Greater Bay Area's economic resilience and its forecast.

### Spatially

It develops in an imbalanced way, high in the central and southern parts and low in the northwest. The spatial pattern of the high-resilience areas is stable, mainly concentrated in the first-tier urban areas. Benefiting from the degree of economic development, industrial structure optimization and economic innovation capability, these high-resilience cities play an essential role in enhancing the resilience of the entire Greater Bay Area through economic connectivity. The space with relatively high resilience has shifted, mainly in Jiangmen and Zhongshan in the west wing of the Greater Bay Area, Hong Kong, and Zhuhai in the south of the Greater Bay Area. These cities have a sound industrial base, but have a high economic risk (e.g., Hong Kong), and are vulnerable to the global economic crisis and their economic resilience fluctuates heavily. The space with relatively low resilience continues to expand, such as Dongguan, Foshan, Zhongshan, and Macau. The industrial structure of these areas is relatively homogeneous, which restricts their economic activities and diversified development, so they need to upgrade and adjust their industries, increase the economic evolutionary power, and improve the level of resilience. The space with low resilience is basically in the periphery. These cities have a poor economic foundation, are lagging behind in industrial restructuring, and are slow to recover from economic crises and public health emergencies once they are hit. Therefore, these cities are the priority target for enhancing the economic resilience of the Greater Bay Area.

### Influencing Factors

The economic resilience of the Guangdong-Hong Kong-Macao Greater Bay Area is significantly correlated with the economic status and economic response, while it is less correlated with the economic pressure resilience. Economic evolutionary power is the key, with a correlation co-efficient of 0.7474. Economic stability and economic recovery capacity are the main focus, while the changes in economic resistance and economic risk should be paid attention to. As for the subfactors, local GDP, industrial value added and urban per capita income have contributed significantly to the increased economic resilience of the Greater Bay Area, reflecting the importance of regional social and economic development, industrial construction and market consumption. At the same time, the positive effects of general public budget revenue and the number of patents applied or granted per 10,000 persons also indicate that the government's ability to manage public health emergencies and the technological means in the epidemic also play a crucial role in the socio-economic operation. Therefore, the foundation of economic development, industrial structure, government regulation and control capacity, and technological innovation capacity are of vital importance to the economic resilience of the Greater Bay Area, and are also important elements in the future resilience enhancement path of the Greater Bay Area.

## Data Availability Statement

The original contributions presented in the study are included in the article/supplementary material, further inquiries can be directed to the corresponding author/s.

## Author Contributions

YZ and BT contributed to the topic selection, development of the framework and writing of the manuscript, and figures and the literature review. ZC contributed to the data analysis, drawings and the tables. HS contributed to the revision of data analysis and drawings. All authors contributed to the article and approved the submitted version.

## Funding

We are grateful for the financial support from project of the 14th five year plan for the development of Philosophy and Social Sciences in Guangzhou in 2021 (No. 2021GZYB22); College students' innovative entrepreneurial training program: Resilience assessment of urban agglomerations in the Guangdong-Hong Kong-Macao Greater Bay Area based on the PSR model (No. S202013902038); School level scientific research project of Guangzhou Xinhua University (No. 2018KYQN001); Study on the spatial pattern, mechanism and management of innovation in the Guangdong-Hong Kong-Macao Greater Bay Area from the perspective of regional synergy. Project of special innovation classes for regular universities in Guangdong Province (philosophy and social sciences) (No. 2020WTSCX136); College students' innovative entrepreneurial training program (No. 201913902171); School level scientific research project of Guangzhou Xinhua University (No. 2018KYZD002); Special research on industry in Longjiang Town, Huilai County (2015HX001).

## Conflict of Interest

The authors declare that the research was conducted in the absence of any commercial or financial relationships that could be construed as a potential conflict of interest.

## Publisher's Note

All claims expressed in this article are solely those of the authors and do not necessarily represent those of their affiliated organizations, or those of the publisher, the editors and the reviewers. Any product that may be evaluated in this article, or claim that may be made by its manufacturer, is not guaranteed or endorsed by the publisher.
